# Mechanistic Effects of Environmental and Medical Low-Dose Radiation Exposure of the Lung

**DOI:** 10.3390/biomedicines14030644

**Published:** 2026-03-12

**Authors:** Stephanie Puukila, James McEvoy-May, Antony M. Hooker, Dani-Louise Dixon

**Affiliations:** 1 College of Medicine and Public Health, Flinders University, Bedford Park, SA 5042, Australia; 2 Medical Sciences Division, Northern Ontario School of Medicine, Sudbury, ON P3E 2C6, Canada; 3 Centre for Radiation Research Education and Innovation, Adelaide University, Adelaide, SA 5005, Australia

**Keywords:** low-dose radiation, lung, linear no-threshold model, adaptive response, radon

## Abstract

Ionizing radiation has been an important tool in medical diagnosis and treatment. While the use of radiation for diagnostic purposes has been successful, clinicians are wary of the possible negative effects radiation may have on the patient. According to the linear no-threshold model, all levels of radiation are considered harmful and there is no safe threshold. However, some studies suggest there may instead be a hormetic response at lower doses typically defined as exposure below 100 mGy, and that low doses may be beneficial as a possible immunomodulatory therapeutic. Therefore, it is increasingly important to understand the effects of exposure to low doses of radiation. The lung is frequently exposed to radiation from both environmental and medical sources. The effects of low doses of radon, the most heavily studied public radiation exposure source, are still contested, as well as the potential risk from medical X-ray imaging and computed tomography exposures during diagnostic procedures. In order to appropriately evaluate the potential risks and benefits of a low-dose exposure, it is necessary to understand the mechanism(s) of action, particularly the role of DNA damage, reactive oxygen species, inflammation and immune response. Here, we review the mechanistic evidence of low-dose radiation exposure effects on the lung in the current literature and discuss the implications of these results on the validity of the LNT model as well as potential hormetic or adaptive responses.

## 1. Introduction

All living organisms are exposed to natural background radiation from both terrestrial and cosmic sources. The average annual dose from this natural background radiation is about 2.4 mSv but varies widely across the planet. As we have evolved in the presence of radiation, an absence of natural background radiation has been shown to have negative consequences [1,2]. It is ubiquitously accepted that high doses of ionizing radiation (>100 mSv) can act as a carcinogen. As radiation passes through a cell, it deposits energy along its track. This energy deposition causes biochemical modifications, including DNA damage. This damage includes double-strand breaks, single-strand breaks and base damage [3,4]. DNA damage triggers repair pathways. Successful repair allows the cell to continue in the cell cycle. However, unsuccessful repair can trigger cell death, while uncorrected repair will generate mutations [5,6]. The efficacy of low-dose radiation (<100 mSv) exposure in cancer induction remains the subject of intense debate. Risk estimates are calculated using the linear no-threshold (LNT) model, which assumes a direct relationship between radiation dose and cancer risk at all doses. The LNT model is based on experimental and epidemiological data from observations made at high doses of radiation exposure, primarily from the atomic bomb survivor cohort [7,8]. Extrapolations of risk estimates based on observations made at high doses are used to estimate risk at lower doses. Therefore, according to the LNT model, all levels of radiation are considered harmful and there is no safe threshold.

The LNT model is used to set recommended exposure dose limits for nuclear energy workers (20 mSv/year) and the general public (1 mSv/year) [9]. This exposure dose is dependent on the type and source of the exposures. Types of ionizing radiation include γ and X-rays, which have relatively low linear energy transfer but higher penetrative capabilities, and α and β particles, which have high linear energy transfer but low penetrative capabilities. High-LET neutrons, when expelled from nuclei, such as during nuclear fission or fusion, have very high penetrative capabilities and can be absorbed by other atoms, creating radioactive isotopes. Exposure can occur from the environment, such as the Earth’s crust (uranium and thorium), biological sources (potassium-40, carbon-14), cosmic rays and medical procedures (diagnostic imaging, radiotherapy). Currently, medical diagnostic procedures contribute to almost half of the annual background exposure dose, depending on geographical region. While the International Commission on Radiation Protection (ICRP) does not recommend a limit on medical radiation exposure [9], concerned physicians may try to restrict the amount of radiation patients receive, as per the principal guideline of radiation safety “as low as reasonably achievable”. Many health care workers, including radiologists, are unsure of the risks or even the doses of diagnostic radiological procedures [10,11]. Therefore, there is a need to increase our understanding of the effects of radiation at diagnostically relevant doses. There is also growing evidence that low-dose radiation can elicit an adaptive response. The adaptive response occurs when a sudden, yet non-lethal, increase in a toxin/stressor in a biological system elicits a stress response, and in turn, stimulates protective mechanisms [12,13]. The adaptive response has been seen in human lung epidemiological studies at X or γ-ray doses below 2 Gy [14]. A review by Scott et al. (2008) found that exposure to diagnostic levels of radiation could reduce the risk of future cancers [15]. Yet, many believe there is not enough evidence to move away from the LNT model to calculate risk at low doses.

Different tissues have different radiosensitivities, which need to be taken into consideration when calculating dose and risk. This is known as the tissue weighting factor (TWF). The lung is in the high carcinogenic risk category, with a tissue factor of 0.12 [9]. Tissue radiosensitivity is likely, at least in part, due to rate of cell turnover. Lung epithelial cells have an average cell turnover time of 30 days [16]. In contrast, the liver, with a TWF of 0.04, has a cell (hepatocyte) turnover time of years [17]. Cells with higher rates of turnover can be ‘hit’ with higher doses before damage (genomic instability/mutation) is observed. It is well established, as reviewed by Olivier and Peikert (2018) [18], that high doses of ionizing radiation to the lung result in pneumonitis and fibrosis. However, the effects of low doses of radiation on the lung (<100 mGy) have not been adequately characterized. While at low doses there appears to be a decrease in radiation’s effectiveness to induce cancer [19], many debate otherwise. Therefore, it is imperative to investigate the low-dose effects, particularly as the lung is a relatively radiosensitive tissue. In this review, we examined the current literature on the effects of low-dose radiation exposure in the lung.

## 2. Materials and Methods

This narrative review was designed to highlight and discuss the mechanistic evidence of low-dose radiation exposure effects on the lung and the implications of these results. We primarily utilized MEDLINE and Web of Science databases for the literature search. Search operators AND/OR were used to combine search terms, which included “low dose”, “radiation”, “lung”, “radon”, “environmental exposure”, “medical exposure”, “in vitro” and “in vivo”. The search was limited to English language articles. Supplementary searches were included from the Flinders University library services. As there are limited data on the specific effects of low-dose radiation exposure on the lung, we expanded our timeline from January 2005 and November 2025. Earlier publications were used for historical context. Google was also used to search public websites for companies, such as commercial Radon Spas. The findings have been summarized in Table 1.

The intention of this review was to adhere to a definition of low-dose radiation exposure of <100 mGy. However, what constitutes ‘low dose’ is dependent on radiation type/source, acute or chronic exposure and whole body or tissue/organ specific exposure. Dose rate is also an important factor to consider, as a single exposure of a high or low dose will induce a different response than if the same total dose is fractionated. This review primarily defined a total, whole-body (or equivalent for in vitro studies) exposure of less than 100 mGy as low and above 100 mGy as high dose, whether exposure was acute or chronic. The exception was lifespan studies, where dose rate was required to be considered as an exact total dose may be unknown. Also, the goal of this review was to include both environmental and medical sources of exposure. Therefore, relevant units of radiation (mGy, mSv, Bq/m^3^) were used when discussing these studies. The primary objective of this review was to summarize low-dose effects in the lung rather than to conduct a quantitative dose–response meta-analysis. Consequently, direct quantitative comparisons across datasets should be interpreted cautiously. Data were interpreted within the context of the respective radiation quality rather than treated as dosimetrically or biologically interchangeable.

## 3. Environmental Exposure to Low-Dose Radiation

The majority of our exposure to radiation is through the environment from naturally occurring sources. According to the United Nations Scientific Committee on the Effects of Atomic Radiation (UNSCEAR), the annual global average effective dose is 2.4 mSv/y [65]. However, there are several geographical high background radiation areas (HBRAs), these include Ramsar, Iran; Kerala, India; Yangjiang, China; and Guarapari, Brazil. In Ramsar, the inhabitants can receive an effective dose of 260 mSv/y; however, cytogenetic studies have shown no significant differences between the people in HBRAs compared with average background areas [66]. An increase in immune response, via an increased cluster of differentiation (CD)69 expression on stimulated CD4^+^ T cells and increased total serum immunoglobulin E, was seen in healthy Ramsar inhabitants compared with average background healthy controls [67]. When lymphocytes from Ramsar HBRA resident volunteers were challenged with a dose of 1.5 Gy γ-radiation, chromosomal aberrations were lower compared with average background areas [66]. Peripheral blood mononuclear cells from residents of HBRAs, challenged with 4 Gy γ-radiation, had a decreased incidence of micronuclei and apoptosis compared with average background controls. While DNA damaged increased, the rate of repair was also increased [68]. Similar results have been observed in Kerala, India, where radiation exposures vary from <1 to 45 mGy/year due to thorium deposits in the beach sand. Micronuclei frequencies were fewer when compared with frequencies from average background controls [69], and DNA repair was more efficient after a high-dose challenge [70]. In Yangjiang, China, where average annual doses of radiation vary from 5.06 to 6.86 mSv/y, studies have found that cancer mortality [71,72] and non-cancer [72] mortality rates were not increased compared with average background areas. This may also be due to more efficient DNA repair and antioxidant activity [73]. Unfortunately, there are currently no reliable epidemiological studies on Guarapari, Brazil, where radiation dose ranges from 3.5 to 10 mSv/y. Also, there have been no studies on the specific effects of living in high background areas on the lung.

Radioisotope exposure also includes accidental background exposure. Lehrer and Rosenzweig (2015) [20] investigated lung cancer incidence in states that have higher background radiation than the average in America due to uranium mining or states downwind of Nevada, where nuclear device testing occurred. These include Arizona, Colorado, Idaho, New Mexico, North Dakota, Oregon, Texas, Utah, Wyoming, Nevada, South Dakota and Washington. Lung cancer incidence in these states was compared with lung cancer incidence in low-impact radiation states. The investigators found lung cancer incidence was significantly lower in high-impact states in both men and women than in normal-impact radiation states [20]. In the early 1980s in Taipei City, Taiwan, steel contaminated with cobalt-60 was accidentally used in the construction of about 1700 apartments, which housed around 10,000 people for between 9 and 22 years. Residents received an individual mean radiation dose of 400 mSv; yet, cancer deaths in this population were 3% of the incidence of spontaneous cancer death in the general Taiwan public [74]. The results of these studies contradict the LNT model and, instead, suggest low levels of radiation exposure may protect against cancers, including lung cancer.

Relatively recently, radioisotopes produced during nuclear fission, released into the environment during nuclear accidents or weapon detonation, have become a source of radiation exposure. Unfortunately, it is difficult to investigate prolonged background exposure in the event of a nuclear accident, such as Chernobyl and Fukushima, as residents in these areas were evacuated and rarely return. To address growing concerns over major accidents in large nuclear power plants and the potential human health effects [75], there has been extensive research on internally deposited radioactive materials using beagle dogs [76,77]. Lung cancer and survival were evaluated in dogs that inhaled various doses of insoluble radioisotopes, which included cerium-144, strontium-90, yttrium-90 and yttrium-91. This was found to be heavily dependent on dose rate rather than the total dose [21,22,23,78]. High dose rates (1–100 Gy/day) of each radioisotope shortened lifespans due to radiation pneumonitis and ‘medium’ dose rates (0.1–10 Gy/day) shortened lifespans due to lung cancer, but low dose rates (<0.1 Gy/day 90Sr, <0.5 Gy/day 144Ce, <0.9 Gy/day 91Y, <8 Gy/day 90Y) did not significantly decrease lifespan compared with non-exposed controls [21,22,23]. The survivors of the A-bomb cohort are the primary source of data for estimating cancer risk using the LNT model. Analysis of the Life Span Study, a research program investigating life-long health effects of the A-bomb cohort, has led some to conclude that there are increased cancer incidents in a linear dose–response analysis [79,80]. Yet, other analyses of the same data disagree [81], and even conclude that there are decreased cancer incidents and prolonged lifespan with low-dose exposure [82]. Possible reasons for these inconsistencies are discussed in more detail below.

Most data available on the effect of environmental radiation exposure on the lung are from radon studies, as radon is responsible for most environmental radiation exposure. Radon is a naturally occurring noble gas that occurs in the decay chain of uranium-238. While radon gas is inert, with a half-life of 3.8 days, it readily decays to produce radioactive radon decay products (RDPs). These RDPs are short-lived and result in the release of α and β-radiation during decay. It is widely stated, due to conclusions drawn from the report ‘Health Effects of Exposure to Radon’ by the Committee on the Biological Effects of Ionizing Radiation (BEIR) VI, that radon is the second leading cause of lung cancer after cigarette smoking [83]. The U.S. Environmental Protection Agency (EPA) has used the BEIR VI report to develop a national action plan to reduce residential radon, and thus lung cancer [84]. Cancer risk from residential radon exposure has been calculated by extrapolating data from 20 epidemiologic studies on uranium miners via the LNT model [83,85]. However, the data are based on exposure to much higher doses than found in residences or workplaces; therefore, its relevance for the calculation of risk at these lower exposure doses has been contested [86,87,88]. Exposure of human lung epithelial A549 cells to 1000 Bq/m^3^, the limit for workplace exposures, showed reduced viability over time [89]. For residential exposures, many studies have concluded that lung cancer risk increased parallel to radon [24,25,26,27,28,29,30,31,32,33,34,35,36,90,91] However, re-analysis of some of these studies have found no such cancer risk of radon exposure at lower doses [37,38]. A case-control study by Thompson et al. (2008) in Massachusetts found contrasting results with other case-control studies and saw a hormetic response with exposures less than 150 Bq/m^3^ [39]. Sandler et al (2006), found no evidence of an increased risk for lung cancer at lower doses of radon exposure [40]. Zarnke et al. (2019) have closely investigated the BEIR VI report and have come to a different conclusion, where “Next to cigarette smoking, high levels of radon combined with cigarette smoking is the second leading cause of lung cancer” [87]. These inconsistencies highlight some of the issues with human epidemiological studies and the LNT model, which are further discussed below.

## 4. Medical Radiation Exposure

In recent years, medical radiation exposure has increased and now contributes an average yearly dose of approximately 0.6 mSv [92]. Therapeutic exposures use very high doses (>1 Gy) of radiation, while diagnostic exposures are considered low dose. Diagnostic procedures include conventional X-rays, computed tomography (CT), fluoroscopy and nuclear medicine. The adult effective dose for each procedure is given in Table 2 and Table 3, as determined by Mettler et al. (2008) using published data from between 1980 and 2007 [93].

Fluoroscopic examinations give individual exposures, similar to lower-end CT scans [94]. The dose is highly dependent on the timing of the exposure [95]. Nuclear medicine uses radiopharmaceuticals to visualize internal structures via γ-radiation produced by radioisotopes [96]. Procedures can range from 0.2 to 40 mSv, as seen in Table 2 [93]. Both fluoroscopy and nuclear medicine procedures are used infrequently [93,94]. X-rays are the most frequently used diagnostic procedure, though the amount of radiation received from X-ray procedures is relatively low, where the average chest X-ray accounts for 0.02 mSv [93]. CT scans can provide more information than conventional X-ray imaging, but doses can vary significantly depending on procedure and location. An abdominal CT is approximately 8 mSv but can vary 5-fold (Table 3) [93]. The use of CT for diagnosis has increased over time. In 1989, CT represented 2% of radiological procedures and contributed 20% to the cumulative effective dose (CED) [97]. In 1998, the figures were 5% and 40% CED, respectively [98], and in 2014, 11% and 82% CED, respectively [94]. Currently, a CT scan can compile an image within a few seconds, thereby significantly decreasing the dose. Yet, there are still efforts to further reduce CT dose. One cohort that this may be particularly true is of pregnant patients in an effort to reduce or prevent exposure on the fetus yet may put the patient at risk of misdiagnosis. A recent audit in Australia found that, of 547 patients, the median cumulative effective dose was 0.02 mSv, with 19.07 mSv the highest dose received. This suggests that pregnant patients are exposed to low cumulative doses of radiation [99]. The concern for all CT scan exposure lies in successfully maintaining the effectiveness to diagnose the patient while also lowering the dose to address the cancer risk concerns. A study by The National Lung Screening Trial Research Team found that screening with CT reduced mortality from lung cancer compared with low-dose radiography due to better detection [100]. Yet some studies indicate that current CT scan usage increases lung cancer risk [41], and postulate that within a few decades approximately 1.5–2% of all cancers in the United States may be the result of CT usage [101]. Recently, Pozzessere et al. (2023) [102] reviewed data from international lung cancer screening trials to estimate radiation-related risk from low-dose CT scans. They found that the effective dose ranges of the trials were 0.2 mSv to 2.36 mSv, below the average CT scan dose and annual global background exposure dose. Yet, the authors still express concerns on exposing healthy individuals to radiation for CT scans and the additional exposure when lesions are detected [102]. Hendrick and Smith (2024) [103] also used data from international lung screening trials to determine the benefit-to-radiation risk ratio. They determined, based on linear no-threshold BEIR VII dose risk estimates, the benefits of lung cancer screening significantly outweigh the harm [103]. Alternatively, it has been argued that the low dose from CT scans can stimulate activated natural protection (ANP) and instead reduce cancer risk [15]. X-ray exposures induce ANP against cigarette-smoking-associated and α-radiation-induced lung cancer in humans [42,43]. When assessing CT scan-induced risk, it is important to ask: does the disease cause the CT or does the CT cause the disease? [104]. Those who receive CT scans must have predisposed clinical justification, while healthy people should not typically be exposed to any medical radiation. Due to the ethical inability to include adequate control groups, comparison between cohorts that receive CT scans and those that do not is itself a significant confounder of CT cancer risk studies.

Interestingly, even with the generally negative perception of radiation exposure, many patients visit radon spas in the United States, Europe and Japan for therapeutic purposes. These ‘spas’ include caves, springs and baths with exposure via inhalation or transcutaneous resorption of radon in water, and are used to, typically, relieve arthritis and chronic inflammatory conditions. In the United States, the use of radon for therapeutic purposes is controversial, and is considered ‘alternative therapy’ and therefore not covered by medical insurance [105]. Only four mines in the United States, near Boulder and Basin, Montana, are used for radon therapy. As there are no medical professionals available at these mines it is up to visitors to choose their ‘treatment’. Only the Free Enterprise Radon Health Mine has posted the typical radon doses in the mine on their website, where radon levels average about 1700 pCi/L (62.9 kBq/m^3^), and range from 700 (25 kBq/m^3^) to 2.2 nCi/L (81.4 kBq/m^3^) [106]. The beneficial claims posted on these facilities’ webpages are based on customer testimonials; however, they do provide links to published studies on the therapeutic effects of radon. In Europe, radon therapy is a more accepted form of treatment, and is available as inhalation exposure, similar to the US, or as baths [107]. The best-known center is at Bad Gastein, Austria, where the air averages approximately 43 kBq/m^3^ (1.2 nCi/L), with a maximal value of 160 kBq/m^3^ (4.3 nCi/L) [108]. While there is some evidence suggesting that radon therapy in the US and Europe can help with chronic pain, not only has the efficacy in lung pathologies not been investigated but the direct effects of these exposures on the lung do not appear to be a concern of the spas administrating them. These centers claim this exposure to radon is safe as it is only “twice the amount of natural background radiation one might receive on an annual basis” [106]. In Japan, where spring water is rich in radium (such as Misasa), the water is used for therapeutic purposes as well as studies on beneficial effects of this therapy [109,110,111,112], including for lung cancer [44]. The Japan Atomic Energy Agency and Okayama University have developed a radon facility to elucidate the mechanism(s) of response to this radon therapy. Despite the lack of any strong scientific evidence on the effects of this radon exposure on the lung, many continue to seek out these alternative treatment locations. Importantly, as stated, positive claims posted on the spa websites come from customer testimonials, not medical professionals, with the intention to promote these spas to other potential users.

Human epidemiological studies are used by regulatory agencies when determining radiation protection. The validity of these epidemiology studies is controversial, and, as discussed previously in this manuscript, may be associated with large biases [113]. Using a different statistical analysis on the same dataset can provide contrasting results [37,38]. Also, it has been stated that epidemiological data below doses of 100 mSv and dose rates of 0.1 mSv/min have low statistical power [114,115]. It is also important to recognize the limitations of case-controlled vs. cohort studies [116]. Case-controlled studies observe an outcome (e.g., cancer) and then identify which patients received the exposure (e.g., radiation). Cohort studies observe the exposure (e.g., radiation) then follow to the outcome (e.g., cancer). While cohort studies are powerful, they require large numbers to observe rare events. In contrast, case-control studies are good at assessing rare events with small patient numbers but can typically have a reporting bias not evident in cohort studies [117]. It has been argued that well-controlled laboratory mechanistic studies are required to complement the epidemiological data to evaluate the effects of low-dose radiation [113,115]. Also, epidemiological studies rarely consider the unique morphology and physiology of the lung, which includes an effective cleaning system [118]. This system is incredibly important for inhalation exposures, particularly radon. In short, this cleaning system consists of two components: a mucus layer and a periciliary layer. The mucus layer traps inhaled infectious and toxic agents, which can include inhaled RDP. The periciliary layer contains embedded cilia that can transport the inhaled dust containing RDP out of the lungs, possibly before the RDP isotopes decay and irradiate the lungs [118,119]. Additionally, in the alveoli, pulmonary surfactants reduce surface tension in the lungs as well as bind and clear foreign particulate matter and pathogens, whereas immune cells, such as macrophages, remove particulate matter as well as clear and recycle surfactants, further enhancing the lungs natural cleaning system [120,121]. It appears that the cleaning system can be affected by temperature, smoke and air pollution. Therefore, it is not possible to accurately calculate an individual dose for the lung epithelium based simply on the airborne radon concentration in the home [118]. These uncertainties further highlight the importance of animal and cell culture studies to enhance our understanding of the cellular and immunological as well as mutagenic effects of low-dose radiation on the lung.

## 5. Mechanism of Low-Dose Radiation Response

With regard to the effects of low-dose radiation on the lung, the most common primary outcome is cancer. Animal and cell culture studies are ideal to investigate the mechanism(s) involved in this response. Though these studies cannot provide direct extrapolations to cancer risk in humans, they provide valuable data on the mechanistic responses to low-dose radiation exposure that are not possible to investigate in clinical trials or epidemiological studies, particularly in regards to lung exposure [19,122,123,124]. Nagashima et al. (2018) observed a possible critical dose for mutagen events induced by multiple DNA double-stranded breaks between 100 and 200 mGy using a hamster cell line [125]. However, studies in mice have shown a suppressive effect of whole-body low-dose exposure (<200 mGy) of γ-radiation on spontaneous [45,46] and artificially induced lung cancers [46,47,48,126]. These responses are proposed to be due to ANP via low-dose radiation-induced DNA damage repair, selective apoptosis and decreased oxidative stress and inflammation [15].

DNA double-stranded breaks are repaired by one of two pathways: non-homologous end joining and homologous recombination. Double-stranded breaks are detected by numerous proteins, and low-dose radiation results in the upregulation and increased activity of many of these DNA repair proteins, as reviewed by Tharmalingam et al. (2019) [127]. The role of the gene ataxia telangiectasia mutated (ATM) has been examined in lung models. Homozygous mutations of ATM are associated with radiosensitivity [128]. In HBE135-E6E7 normal human lung epithelial cells, exposure to a single X-ray dose of 75 mGy increased ATM and thereby AKT phosphorylation. This was not seen in A549 human adenocarcinoma alveolar epithelial cells [49]. Yet, in A549 cells exposed to 200 mGy from γ-rays, increased ATM kinase protein was observed, likely due to observed low-dose hyper-radiosensitivity of the immortalized cancer cell line [50]. Knockout of ATM in C57Bl/6 mice resulted in increased DNA double-stranded breaks in lung tissue, as indicated by increased 53BP1, when exposed to a whole-body dose of 100 mGy daily for up to 10 weeks [51]. Double-stranded breaks were observed in primary human fibroblasts from the lung after 200 mGy exposure. The majority of these were repaired 24 h post-exposure. Interestingly, in cells exposed to a much lower dose of 1.2 mGy, double-stranded breaks remained unrepaired for many days. However, when these cells were allowed to proliferate after exposure, the number of double-stranded breaks was decreased. The authors state that this was likely due to the elimination of unrepairable cells via an observed increase in apoptosis. This was also seen when cells exposed to 200 mGy were allowed to proliferate [52]. Low doses of low LET radiation activated protective apoptosis responses that selectively removed precancerous and irregular cells [129,130,131,132]. This did not occur after doses of high LET α-radiation but did seem to occur after combined exposure to α and γ-irradiation, where it can be assumed that selective apoptosis was activated by the γ-ray component of the dose [43,130,133]. Scott B (2007) proposed that the reduction in expected lung cancers, based on the LNT model, after chronic low dose radiation exposure [43], such as that seen in the cobalt-60-contaminated apartment buildings in Taiwan [74] or in the laboratory [45,46,47,48,126], could be due to protective apoptosis [43]. These DNA repair and selective apoptosis responses have only been observed in cell and animal models, and the role in human lung cancer has yet to be confirmed. As reviewed by Bauer (2007), many studies have shown selective apoptosis induction in transformed cells to be through reactive oxygen species (ROS)-mediated signaling [134]. Increased ROS has also been implicated in DNA repair response [135]. These responses are likely the same in low-dose radiation exposures.

The direct effect of radiation on DNA molecules accounts for 30–40% of DNA lesions. The other 60–70% is due to the generation of free radicals through the radiolysis of intercellular water forming ROS [5,136,137]. ROS-induced cellular damage may continue for days and months after the initial exposure, contributing long-term to genomic instability [136]. ROS generation from low doses of radiation induce adaptive responses due to increased antioxidant capacity [136,138,139], which results in cells resistant to further insult [139], as well as attenuating DNA damage [136,138,140]. Nuclear factor erythroid 2-related factor 2 (Nrf2) regulates oxidative equilibrium via the production of downstream antioxidants and plays a role in low-dose radiation-induced oxidative response. In A549 cells exposed to 50 mGy, Nrf2 and downstream heme oxygenase-1(HO-1) protein were increased. These cells had greater survival after a secondary challenge dose of 750 mGy than cells exposed to 750 mGy alone. Inhibition of Nrf2 or HO-1 suppressed this 50 mGy induced radio resistance, indicating this response is Nrf2-dependant [53]. In HBE135-E6E7 cells, mRNA and protein levels of Nrf2-dependent antioxidant factors HO-1 and NAD(P)H quinone dehydrogenase 1 (NQO-1) were increased after a single exposure of 75 mGy X-rays, as well as the nuclear fraction of Nrf2. This increase in Nrf2 and downstream products was dependent on the increased phosphorylation of ATM, AKT, and glycogen synthase kinase 3 beta (GSK-3β) [49]. These pro-survival and antioxidant responses were ROS-dependant. In HBE135-E6E7 cells, 75 mGy induced low levels of ROS and resulted in the activation of ATM, and thus, increased Nrf2 and downstream antioxidant products [49]. In A549 cells exposed to 50 mGy, superoxide was increased by 16.5% compared with non-irradiated control cells [53]. This increase in ROS induced autophagy, made evident via increased LC3-II. When ROS scavenger N-acetyl-L-cysteine (NAC) was added, the low-dose radiation-induced increase in autophagy was no longer observed. Radio resistance from an additional exposure of 750 mGy was also no longer observed. The suppression of Nrf2 or HO-1 in 50 mGy-exposed A549 cells also resulted in decreased autophagy and a loss of radio resistance to the subsequent higher doses [53]. Antioxidant responses have also been observed in lung tissue of some animal models of low-dose radiation exposure but, interestingly, not all. When Sprague Dawley rats were exposed to a whole-body dose of 2, 20 or 200 mGy X-rays, there was no observable change in catalase or superoxide dismutase 2 (SOD2) in the lung compared with sham controls up to 24 h post-exposure [54]. However, increased gene expression of glutathione peroxidase was observed in C57Bl/6 mice after a whole-body γ-ray exposure of 200 mGy until 3 days post-exposure [55]. Also, reduced glutathione levels were increased in the lungs of Balb/c 24 h post-exposure of a whole-body γ-ray dose of 100 or 250 mGy, yet there was no change in SOD, catalase activity, glutathione peroxidase or glutathione reductase activity [56]. Even without the antioxidant response, it appears that the amount of ROS generated from low-dose radiation was too low to cause damage in these models. In Sprague Dawley rats exposed to a whole-body dose of 2, 20 or 200 mGy X-rays there was also no change in 4HNE, a stable by-product of lipid peroxidation, in the lung compared with sham control lungs [54]. Whole-body doses of 100 mGy and 500 mGy γ-rays also did not alter lipid peroxide levels in the lungs of Balb/c mice. Interestingly, lipid peroxide levels were increased by 38% 12 h after 250 mGy exposure but returned to control values within 24 h [56]. Alternatively, normal lung fibroblasts exposed to fractionated X-ray doses of 1 mGy, up to a total of 460 mGy, resulted in the persistent accumulation of mitochondrial ROS, leading to stress-like responses, including increased mitochondrial mass, decreased cellular levels of glutathione, and genomic instability. Treatment with NAC prevented these responses [141]. While these results indicate a stress response due to increased ROS via low-dose radiation exposure, Azzam et al. (2012) have argued it may also represent adaptive responses that can be protective against an additional insult [136]. Pre-treatment of HBE135-E6E7 cells with a single exposure of 75 mGy resulted in lower ROS levels when exposed to a subsequent dose of 5 Gy compared with cells exposed to 5 Gy alone [49]. While there appears to be some beneficial responses, if and how these responses translate to human lung cancer risk is unknown. Though the full mechanistic response(s) is not yet clear, it is evident that the ROS response to low-dose radiation is dependent on dose, dose rate and cell/strain type.

During inflammation, ROS are produced by innate immune cells via oxidative bursts. High levels of ROS initiate the inflammatory response and induce pro-inflammatory cytokine generation. In turn, these pro-inflammatory cytokines are involved in the generation of ROS via a positive feedback loop [142]. Chronic inflammation has been implicated in genetic instability and tumor formation [143]. In beagle dogs, the inhalation of β-emitting radionuclides at a high dose per cell turnover time induced chronic inflammation, cell killing and tissue disorganization/damage resulting in lung cancer. At lower doses per cell turnover time, these effects were not observed and did not result in lung cancer rates higher than the controls [78]. Low-dose radiation is believed to induce anti-inflammatory response and promote immune functions [12,140]. In a murine model of asthma, where airways were challenged with ovalbumin, low-dose rate chronic γ-irradiation (0.554 or 1.818 mGy/h) for 24 days after initial sensitization significantly decreased cell infiltration into the lung, methacholine responsiveness, and the levels of immunoglobulin E, interleukin IL4, IL-5 and matrix metalloproteinase-9. Airway inflammation and mucus production were also attenuated [57]. Additionally, the authors found that the mechanism in which radiation exposure ameliorates asthma-related progression was macrophage-dependent, where M2 infiltration in the lung was significantly inhibited. Further, Type 2 T helper cytokine secretion (specifically IL-4 and IL-13) in M2 macrophages was decreased in response to low-dose radiation exposure [144]. In a healthy rat model, a single whole-body exposure of 2, 20 or 200 mGy X-rays did not appear to elicit an inflammatory response in the lung up to 24 h post-exposure. Interestingly, in the spleen, cellular apoptosis was decreased after exposure compared with sham animals [54].

ROS are also involved in the control of cell proliferation, particularly hydrogen peroxide and superoxide [145,146]. Specifically, low levels of ROS induce growth, but higher concentrations induce cell death [146]. Similar results have been observed in radiation exposure, where high doses induce cell death but low doses may stimulate proliferation [58,147]. Cell cycle control is critically linked to the initiation of cell proliferation. Radiation-induced ROS exert direct or indirect activation of cellular response pathways and regulatory proteins that control cell cycle checkpoints at G0/G1, S and G2/M phases [147]. Low-dose radiation exposure of 50 mGy enhanced the number of S-phase cells in healthy lung fibroblasts [59], as well as increased overall the number of cells [58,59,60]. Further investigation of the specific pathway(s) involved in this response indicated that low-dose radiation exposure stimulated phosphorylation and activation of MAPK/ERK pathway proteins, including ERK [59,60], MEK, Raf [58,59], and p38, and downstream effectors, such as Elk-1, p90RSK, and ATF-2 [60]. Low-dose exposure also stimulated phosphorylation and activation of PI3K/AKT pathway proteins, including AKT and PDK-1 [58,59]. Similarly, lung epithelial cells exposed to 75 mGy had increased phosphorylation of AKT, ATM, as well as GSK-3β, and increased CDK4, CDK6, and cyclin D1 expression, indicating a likely pathway for the observed increased induction of S-phase progression and increased cell proliferation [49]. These studies also revealed that p53, p21 and JNK were not activated by this low-dose exposure but could be following high-dose exposure of 2 Gy, antagonizing the proliferative effect and resulting in cell cycle arrest [58,60]. There are few studies of radiation-induced changes in proliferation in the lung, and these few studies are in exposure of single cell lines, which may not represent the response in multiple cell types and tissues.

**Figure 1:**
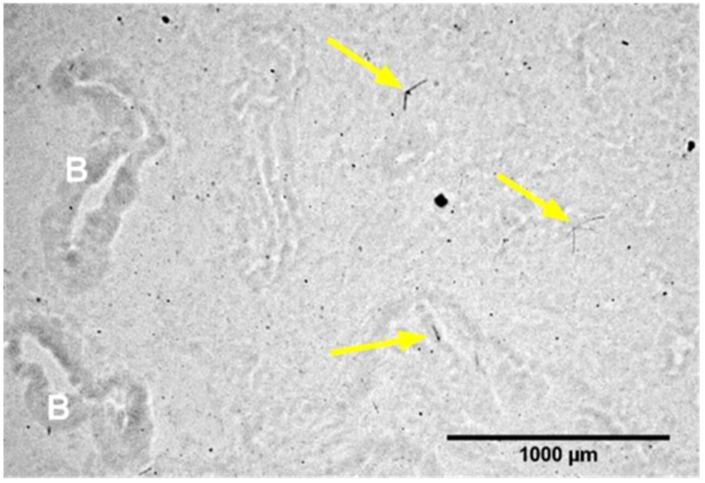
Alpha particle emission of individual radon decay progenies in an adult male Sprague Dawley rat lung after 1000 Bq/m^3^ continuous exposure for 18 h. “B” identifies lung bronchioles. Arrows identify alpha tracks. Originally published in Radiation Research (2025) [62]. Used with permission © 2026 Radiation Research Society.

Cigarette smoke is ubiquitously known to be the leading cause of lung cancer. As cigarette smoke elicits chronic inflammation in the lung, inhibiting this response may be an effective cancer-preventative approach. HFL1 human fibroblasts stimulated by benzo[a]pyrene diol epoxide, a carcinogen from cigarette smoke, increased the secretion of inflammatory mediators IL-6, chemokine CXC ligand (CXCL)1 and CXCL5 via NF-κB activation. Pre-exposure of 90 mGy γ-ray photons suppressed the secretion of cytokines by inhibiting the activation of NF-κB [61]. As with cigarette smoke, fine particulate matter (PM2.5) found in air pollution can cause chronic inflammation in the lung. This is also of concern when combined with radon exposure. Radon decay products, which result in the release of α, β and γ radiation, are charged, and therefore readily attach to small airborne particles. These particles can then be inhaled and deposited in the lung [148]. Recently, a study on radon exposure in a healthy rat model confirmed the presence of α particle emissions of individual radon decay progenies in rat lung of 1000 Bq/m^3^ exposure for 18 h (Figure 1). Prolonged, continuous exposure (4 × 90 h) of radon gas at 1000 Bq/m^3^ resulted in changes in respiratory mechanics and expression of H2AX in the lung [62]. Additionally, the presence of radon decay products is likely to be, at least partially, responsible for the possible synergistic effect of radon and PM2.5 recently observed [149]. Interestingly, in a mouse model of lung damage, a low-dose exposure of 50 mGy alleviated pulmonary inflammation by preventing a PM2.5-induced increase in expression of IL-1, IL-6 and TNF-α mRNA [63], though further evaluation of this response is needed.

As cancer therapy, including chemotherapy or radiotherapy, results in immunosuppression, the effects of low-dose radiation in the immunocompromised patient is a predominant concern [150]. Zhou et al. found that 75 mGy X-ray exposure alone inhibited tumor growth and prolonged survival in a mouse model of Lewis lung cancer. The radiation exposure led to an increase in activated T cells and natural killer cells, increased the cytotoxicity of splenocytes, and increased the infiltration of T cells into the tumor tissues. When the immune function was impaired by the pre-treatment of a high dose of 1 Gy, these beneficial responses were not observed. Yet, when the low dose was administered before the high-dose exposure, tumor growth was again inhibited, due to hormesis and/or adaptive responses [48]. Nowosielska et al. (2010) [126] analyzed the epidemiological and experimental evidence of low-dose exposure to X- or γ-rays on suppression of the development and progression of tumors and metastasis. It was found that low-dose exposure stimulated anti-neoplastic functions of the immune system [126], demonstrating that the immune system plays a pivotal role in low-dose radiation-induced tumor suppression.

It is well established that reduced fetal growth, regardless of stimulus, negatively impacts lung development [151,152,153,154,155]. One environmental stimulus that has yet to be explored is ionizing radiation exposure during pregnancy. For mice, gestational day 15 is a critical time point for the development of the bronchus and bronchioles and the connection of the pulmonary blood supply via peripheral capillaries [156,157]. McEvoy-May et al. (2020) [157] investigated the respiratory development of mice following exposure in utero. Pregnant C57Bl/6 mice were irradiated at gestational day 15 at 50 mGy, 300 mGy, or 1000 mGy γ-radiation. While exposures to 1000 mGy significantly reduced the growth of the pups compared with sham animals, there was no overall effect of ionizing radiation on the respiratory development [64]. Evidently, significant work is required in this model before making any tangible conclusions.

## 6. Discussion

Here, we endeavored to review the existing literature on the effects of low-dose radiation exposure on the lung. Using the LNT model [19], there remains some cancer risk at low doses. An exposure of 100 mGy of 100 kV X-rays results in 100 hits per cell [122,123]. Even a single track of radiation through a cell is believed to induce unique, complex DNA damage [124]. There have been numerous reviews re-evaluating the LNT model with regards to low-dose radiation [15,127,158,159,160]. Some criticism is of the model itself, such as the extrapolation of data at high doses or determining biological risk based solely on the number of cells hit per exposure [127]. Further, these studies potentially highlight the role of hormesis, adaptive response, bystander effects and genomic instability at lower doses [15,127,158,159,160]. Older papers analyzed in the literature provide evidence that both supports and does not support alternative concepts to the LNT model, such as radiation hormesis, at low doses but with primarily animal and epidemiology studies [126,161]. More current reviews included in the literature utilize improved techniques to investigate molecular responses that have additionally provided evidence of potential adaptive responses or hormesis at low doses [127,158]. Most of the existing literature and reviews on low-dose radiation exposure and the role of the LNT model discuss general responses, with few focusing on tissue- or organ-specific exposures. This is the first to focus on the lung-specific response to low-dose radiation exposure.

**Figure 2:**
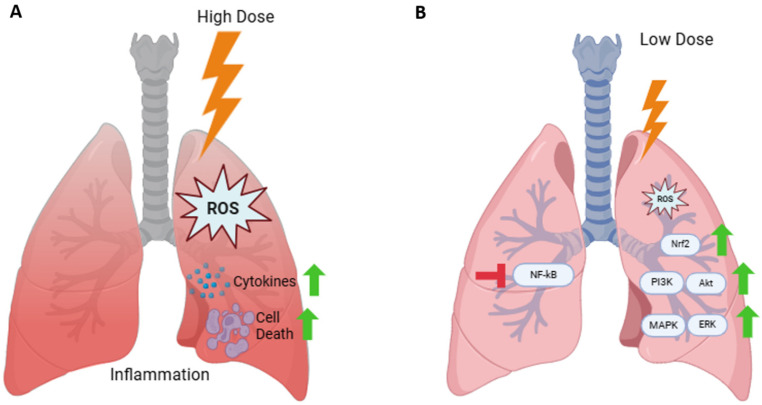
The observed response in the lung induced by (**A**) high (>100 mGy)- or (**B**) low (<100 mGy)-dose radiation exposure based on the current literature. High-dose exposure induces high levels of increased ROS, resulting in increased cytokine expression, cell death and inflammation. Low-dose exposure induces low levels of increased ROS, resulting in NfκB inhibition and increased expression of MAPK/ERK and PI3K/Akt pathways and Nrf2 (Created in BioRender. Puukila, S. (2026) https://BioRender.com/nje3nuy).

The lung is considered radiation sensitive and is in a relatively high carcinogenic category [9], likely, at least, partly due to a high cell turnover rate [16]. Additionally, the lung is a relatively frequently exposed internal organ, mainly due to radon inhalation and medical diagnostics. As described in this review, there is evidence to suggests that at low doses the response in the lung is similar to those previously described [127,158,159,160], including DNA damage repair, selective apoptosis and decreased oxidative stress and inflammation. We have summarized the findings of this review in Figure 2. Interestingly, a recent review by Chaurasia et al (2024) provided a similar summary of cellular responses to high (>100/200 mGy)- and low (<100/200 mGy)-dose exposure [159], further signifying that the response in the lung is similar to other exposure analyses.

There are a number of limitations with this descriptive review that need to be discussed. The epidemiology data presented here at low dose ranges posses a number of issues including dose uncertainties and low statistical power. The molecular data are primarily from animal and cell models. While these can provide valuable information on radiation exposure response, they do not present a direct extrapolation of human lifetime cancer risk, particularly with regards to lifetime cancer risk and low-dose radiation exposure during childhood. Here, we kept the focus primarily on exposures during adulthood, but age is an important factor to consider. It is understood that developmental age and tissue sensitivity of pediatric patients can result in different responses to low-dose radiation exposure. Due to this, there remain concerns of when to utilize medical diagnostics for pediatric patients. Even with advances in imaging at lower and lower doses, parents still face uncertainty. Therefore, further research is needed to allow doctors and parents to make informed decisions on the risks and benefits of diagnostic imaging with radiation. While most of the data presented here do not support an LNT model, we are currently not in a position to suggest an alternative model as a replacement with regards to radiation protection policy.

## 7. Conclusions

Ionizing radiation exposure is increasingly frequent, from both environmental and medical sources. This has been a cause of concern for many medical professionals and members of the public. The lung is a particularly radiosensitive organ that is frequently exposed to radiation from both environmental and medical sources; therefore, it is crucial to adequately understand the response in the lung to radiation exposure. The linear no-threshold model is currently used to estimate risk at all levels of ionizing radiation, even low. Radon exposure is the most relevant radiation exposure to the lung. While there is an abundance of epidemiological studies on residential radon exposure, the leading conclusion of “radon exposure is the second leading cause of lung cancer” is still heavily contested. In fact, many have observed the therapeutic potential of radon exposure. Analysis of the mechanism(s) of action can provide the necessary insight to provide appropriate conclusions (summarized in Figure 2). As the literature indicates, DNA damage, ROS, inflammation and the immune response play critical roles in low-dose radiation response in lung tissue and cells. Interestingly, much of these data suggest possible hormetic or adaptive responses at low-dose exposure rather than increased risk. Also, it is important to consider dose rate when estimating radiation risk. Still, there remains a lack of sufficient evidence to argue the validity of using the LNT model to estimate risk in the lung at low doses.

## Figures and Tables

**Table 1:** Study type, radiation dose ranges, biological endpoints, or dose–effect relationships.

Study Design	Experimental Model	Radiation Dose(s)	Biological Endpoints	Ref.
Epidemiology	U.S.A.	Total background	↓ lung cancer with high total background	[20]
In vivo lifetime	Beagles	<0.1 Gy/day 90Sr, <0.5 Gy/day 144Ce, <0.9 Gy/day 91Y, <8 Gy/day 90Y	no Δ lifespan from control	[21,22,23]
Epidemiology	Meta-analysis	Radon 150 Bq/m^3^	↑ RR (1.14 [95% CI = 1.0–1.3])	[24]
Epidemiology	Case-control	Radon 148 Bq/m^3^	↑ OR (1.79 [95% CI = 0.99–3.26])	[25]
Epidemiology	Collaborative analysis of individual data from case-control studies			[26,27,28,29,30,31,32,33,34,35,36,37,38,39,40,41,42,43,44]
In vivo	♀ A/J mice	γ WBI x6 biweekly 100 mGy	↓ spontaneous foci in the lung	[45]
In vivo	♀ WHT/Ht mice + tumor cell injection	X-ray 0.15–0.20 Gy	↓ incidence of lung metastasis	[46]
In vivo	♀ A/J mice + cigarette smoke carcinogen injection	γ WBI x6 biweekly 100 mGy	↓ lung adenomas	[47]
In vivo	♀ C57BL/6 mice + lung cancer cell transplant	X-ray WBI LDR x4 75 mGy HDR x4 1 Gy	LDR-activated T & NK cells, ↑ splenocyte cytotoxicity & T cell tumor infiltration	[48]
In vitro	A549 cells HBE cells	X-rays 20–100 mGy	HBE—↑ cell proliferation ↑ antioxidants no Δ in A549 cells	[49]
In vitro	A549 cells	γ-ray irradiation 200–500 mGy	↑ ATM kinase protein	[50]
In vivo	ATM^+/−;−/−^ C57BL/6 mice	X-ray WBI 100-mGy x1 daily 10 weeks	↑ cell specific DNA ds breaks in lung parenchyma	[51]
In vitro	Human lung fibroblasts	X-ray 1–200 mGy	↑ DNA ds breaks; repair in 24 h at 200 mGy, not at 1.2 mGy	[52]
In vitro	A549 cells	α particle 5cGy	radio resistance to 75cGy α particle	[53]
In vivo	Sprague Dawley rat	X-ray WBI 2–200 mGy	no Δ in lung	[54]
In vivo	C57Bl/6 mice	γ-ray WBI 200 mGy	↑ glutathione peroxidase	[55]
In vivo	Balb/c mice	Whole-body doses of 100 mGy and 500 mGy γ-rays	no Δ lung lipid peroxide	[56]
In vivo	♀ C57BL/6 mice OVA asthma model	γ-irradiation 0.554 or 1.818 mGy/h for 24 days	↓ lung inflammation, bronchoconstriction	[57]
In vitro	CCD-18Lu cells	γ-irradiation 0.05 Gy	↑ cell proliferation, Raf & Akt; no Δ phospho-p53, p53, p21	[58]
In vitro	Human embryonic lung fibroblast 2BS cells lung cancer NCI-H446 cells	X-rays 20–75 mGy	2BS cells ↑ cell proliferation phosphorylation ERK, MEK, and Raf and AKT H446 cells no Δ	[59]
In vitro	CCD 18Lu cells	γ-irradiation 0.05 Gy	↑ cell proliferation, activated ERK1/2 and p38 no Δ Micronuclei frequencies or JNK1/2	[60]
In vitro	HFL1 human fetal lung fibroblast + cigarette-smoke carcinogen benzo[a]pyrene diol epoxide (BPDE)	γ-irradiation 90 mGy	↓ NF-κB activation proinflammatory cytokines IL-6, CXCL1, CXCL5	[61]
In vivo	♂ Sprague Dawley rats	Radon 400 and 1000 Bq/m^3^ 18 h, 90 h, 2 × 90 h, or 4 × 90 h	↑ H2AX expression 1000 Bq/m^3^ following 4 × 90 h exposure; no Δ lung physiology or immunology	[62]
In vivo	♂ C57BL/6 mice	X-ray WBI x9 50 mGy + PM2.5 group (25 mg·kg^−1^ body weight)	↓ Toll-like receptors induced MyD88/NF-κB ↓ M1 polarization of alveolar macrophages, inflammatory cytokines (IL-1, IL-6 and TNF-α) ↑ anti-inflammatory cytokines (IL-4, IL-10 and TGF-β), TLR1, TLR2	[63]
In vivo	Pregnant C57Bl/6 mice	γ WBI gestational day 15 0 (sham), 50, 300 or 1000 mGy	no Δ in respiratory outcomes in pups at 16–17 weeks	[64]

↓ = decrease, ↑ increase, WBI = whole-body irradiation, LDR = low-dose radiation, HDR = high-dose radiation, ♀ = female, ♂ = male, A549 = human lung adenocarcinoma, HBE = normal lung epithelial cells, CCD-18Lu = human lung fibroblast, IL = interleukin, TNF = tumor necrosis factor, TLR = toll-like receptor, PM2.5 = fine inhalable particles with diameters ≤ 2.5 micrometers.

**Table 2:** Effective doses from various nuclear medicine exams.

Examination	Effective Dose (mSv)
Nuclear medicine	
Brain (^18^F or ^99m^Tc)	5.7–14.7
Thyroid scan (^99m^Tc or ^123^I)	1.9–4.8
Cardiac stress (^99m^Tc or ^201^Tl)	9.4–40.7
Lung ventilation/perfusion (^99m^Tc)	0.2–2
Renal (^99m^Tc)	1.8–6.3
Bone (^99m^Tc)	6.3

^18^F = fluorine 18, ^99m^Tc = Technetium 99m, ^123^I = Iodine 123, ^201^Tl = Thallium 201; adapted from [93].

**Table 3:** Effective doses from various diagnostic radiation modalities including conventional X-rays, computed tomography and fluoroscopy.

Examination	Average Effective Dose (mSv)	Values Reported in the Literature (mSv)
Conventional Radiography		
Skull	0.1	0.03–0.22
Cervical spine	0.2	0.07–0.3
Thoracic spine	1	0.6–1.4
Lumbar spine	1.5	0.5–1.8
LAT chest	0.1	0.05–0.24
AP chest	0.02	0.007–0.05
Abdomen	0.7	0.04–1.1
Pelvis	0.6	0.2–1.2
Hip	0.7	0.18–2.71
Other extremities	0.001	0.0002–0.1
Computed Tomography		
Head	2	0.9–4.0
Neck	3	NA
Head and neck angiography	5	0.8–19.6
Chest	7	4–18
Thoracic angiography	15	13–40
Abdomen	8	3.5–25
Pelvis	7	3.3–10
Abdominal angiography	12	4–48
Spine	6	1.5–10
Coronary angiography	15	7–57
Fluoroscopy		
Intravenous urography	3	0.7–3.7
Upper gastrointestinal series	6	1.5–12
Barium enema	8	2–18

mSv = millisieverts, LAT = lateral position, AP = anteroposterior position, NA = not available; adapted from [93].

## Data Availability

No original data was generated for this descriptive review. Citations can be found via MEDLINE and Web of Science databases or Google.

## Abbreviations

The following abbreviations are used in this manuscript:

Akt	Protein Kinase B
ANP	Activated Natural Protection
ATM	Ataxia Telangiectasia Mutated
BEIR	Biological Effects of Ionizing Radiation
CD	Cluster of Differentiation
CED	Cumulative Effective Dose
CT	Computed Tomography
CXCL	CXC ligand
DNA	Deoxyribonucleic Acid
EPA	Environmental Protection Agency
ERK	Extracellular Signal-Regulated Kinase
GSK-3β	Glycogen Synthase Kinase 3 Beta
HBRA	High Background Radiation Areas
HDR	High-Dose Radiation
ICRP	International Commission on Radiation Protection
IL	Interleukin
LDR	Low-Dose Radiation
LNT	Linear No-Threshold
MAPK	Mitogen-Activated Protein Kinase
NAC	N-acetyl-L-cysteine
NF-κB	Nuclear Factor Kappa B
NQO-1	NAD(P)H Quinone Dehydrogenase 1
Nrf2	Nuclear Factor Erythroid 2-Related Factor 2
PI3K	Phosphoinositide 3-Kinase
PM	Particulate Matter
RDP	Radon Decay Product
ROS	Reactive Oxygen Species
SOD2	Superoxide Dismutase 2
TLR	Toll-Like Receptor
TWF	Tissue Weighting Factor
UNSCEAR	United Nations Scientific Committee on the Effects of Atomic Radiation
WBI	Whole-Body Irradiation
